# Ultrasonographic measurements of the prostate gland in castrated adult dogs

**DOI:** 10.1186/s13028-022-00634-1

**Published:** 2022-07-08

**Authors:** Femke Bosma, Saffiera Wijsman, Simone Huygens, Maartje Passon-Vastenburg

**Affiliations:** 1 Radiology Department, AniCura Medisch Centrum Voor Dieren, Isolatorweg 45, Amsterdam, 1014 AS The Netherlands; 2 Institute for Medical Technology Assessment, Erasmus University Rotterdam, Rotterdam, The Netherlands

**Keywords:** Castrated canines, Prostatic dimensions, Ultrasonography

## Abstract

**Background:**

The dimensions of the prostatic gland in castrated adult dogs, as assessed by ultrasonography, is currently not yet reported in veterinary literature. The current study was aimed at reporting the prostatic dimensions in castrated dogs and investigate the relationship between the dogs’ body weight and prostate size. A second aim of the study was to investigate whether there was a relationship between the dogs’ age and prostate dimensions. A prospective, single-centre, observational study was conducted and 72 privately owned, adult, male castrated dogs with a range of breeds and ages met the final inclusion criteria. The subjects were divided into three categories based on body weight.

**Results:**

A Kruskal–Wallis test found prostatic length and prostatic depth in the longitudinal orientation to be significantly different among the 3 categories (P < 0.005), with an increase in both prostatic length and prostatic depth with increasing body weight. Linear regression of the data set provided comprehensive formulas calculating prostatic length and depth based on the body weight of the dog (r^2^ of 0.69 and 0.53 for prostatic length and depth respectively). Kendall’s Tau rank test showed no correlation between dogs’ age and prostate dimensions (P > 0.100).

**Conclusions:**

The current study is the first to provide a comprehensive, weight-based reference for the canine prostate gland of castrated dogs when assessed on ultrasonography.

## Background

Ultrasonography is a widely used method for assessing the prostate gland of male dogs [1–3]. Previous anatomical, histological and imaging studies have illustrated a clear relationship between the reproductive status (intact or castrated) and the prostatic size in male dogs [4–8]. Furthermore, in intact male dogs a relationship between the prostatic dimensions and both the age and body weight of the dog has been illustrated [4, 6]. Reference values have been published for the dimensions and estimated volume of the prostate gland as measured with ultrasonography in healthy intact male dogs [9, 10]. However, no such reference ranges have been published for castrated male dogs. A recent study investigating the application of contrast-enhanced ultrasonography of the prostate gland in castrated canines reported no correlation between prostatic volume and body weight [11]. However, another recent study concerning the prostatic measurements of castrated canines on computed tomography (CT), reported a positive correlation between body weight and linear measurements of the prostate gland [12]. These contradictory results are noteworthy and invite to further investigate a possible relationship between body weight and prostatic dimensions.

Prostatitis and benign prostatic hyperplasia are generally less common in castrated dogs than in intact dogs, however, prostatic neoplasia has a higher incidence in castrated male dogs compared to intact male dogs [13, 14]. Prostatomegaly is a common finding in prostatic neoplasia, thus illustrating the important clinical application of reference values for prostatic dimensions in castrated male dogs. Other reported ultrasonographic abnormalities associated with malignancies of the prostate include an abnormal shape, mineralization of the parenchyma, invasion into local tissues and regional lymphadenopathy [15, 16].

The work of Atalan et al. [10] indicated that there was no significant correlation between age and prostatic size in castrated dogs, as opposed to intact male dogs in which a positive correlation between age and prostate size was demonstrated.

The aim of the current observational study was to supply a comprehensive reference for the dimensions of the prostate gland of castrated male dogs, free of current or past disease concerning the lower urogenital system as assessed with ultrasonography. A second aim of the study was to assess the effect of age on the prostatic size in castrated dogs.

We hypothesized that there would be a positive correlation between body weight and prostatic dimensions in castrated adult canines. We expected there to be no effect of age on the prostatic dimensions in this population, in line with a previous study [10].

## Methods

### Animals

A prospective, observational study was performed at a private referral companion animal hospital (AniCura-MCD, Amsterdam, The Netherlands) from September 2020—March 2021. The sample size was based on convenience sampling of eligible dogs who presented to our clinic within the study timeframe. Privately owned, adult, male castrated dogs, who presented to our clinic and were undergoing a complete abdominal ultrasonographic examination, were prospectively recruited. Informed consent by the owners was not obtained, as the assessment and measurement of the prostate gland is part of the routine abdominal ultrasonographic examination at the hospital and data used for this study are anonymized. Use of patient data is included in the General Terms and Conditions of the clinic. The use of patient data was approved by the clinic director.

### Inclusion and exclusion criteria

Inclusion criteria for the study subjects were as follows: the dog was presented for clinical signs not related to the lower urogenital system (e.g. no signs of dysuria, pollakiuria, hematuria, tenesmus), the medical history of the dog recorded no diseases related to the lower urogenital system, the dog was 12 months of age or older and the prostatic gland appeared within normal limits during the ultrasonographic examination. A normal prostate gland was defined as a regular shaped, ovoid (i.e. length exceeding height) and homogeneously hypoechoic (compared to adjacent fat) structure. A prostate was considered abnormal when one or more of the following ultrasonographic findings were found: irregular shape, asymmetrical enlargement, heterogeneous echotexture, focal lesions, mass lesions or cavitation. Even though parenchymal inhomogeneities are described as an incidental finding in older castrated dogs, for the purpose of this study these individuals were excluded [17].

Exclusion of a recruited dog occurred if upon ultrasonographic examination there was evidence of disease related to the urinary bladder, urethra or prostate gland, if a clear image of the prostate gland was not accomplished due to patient factors and/or artifacts (complete or partial intrapelvic location of the prostate with acoustic shadowing of the pelvis preventing the identification of the organ margins, poor visualization of organ margins due to suboptimal ultrasound beam penetration (e.g. due to patient size and body condition score or cutaneous lesions), lack of urinary bladder filling resulting in loss of acoustic window) or if pertinent medical information was missing from the available patient history (e.g. approximate age at castration).

Exclusion of a dog occurred either during the initial ultrasonographic examination or after reviewing archived medical records and DICOM images (Digital Imaging and Communications in Medicine). From the medical history the following information was gathered and entered into a spreadsheet using Microsoft Excel: current age (in months), body weight (in kg), breed and body condition score (using a 1 through 9 scale as described by the WSAVA). Final subject selection was conducted by the first author (FB).

### Data recording

The images used for prostatic measurements were obtained by an European College of Veterinary Diagnostic Imaging (ECVDI) certified radiologist or by either a 1st or 2nd year ECVDI radiology resident under veterinary radiologist supervision. During the examination both still images and cine loops of the prostate gland were obtained and saved onto the hospitals Picture Archiving and Communication System (EasyImage, VetZ GmbH, Isernhagen, Germany) in DICOM format. The ultrasonographic examination was conducted in a standardized fashion, as per protocol of the hospital, in awake animals with a 4–15 MHz linear array or 6–10 MHz convex array probe (MyLab EightVET, Esaote S.p.A. Genoa, Italy).

The prostatic measurements were all performed by the same author (FB), either on archived images or on freeze images from archived cine loops, at the discretion of the author. Due to the nature of the study design, the author had access to all available patient information at the time of measurement acquisition.

**Fig. 1:**
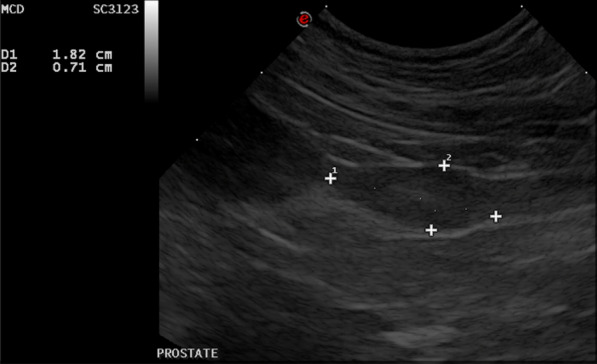
Longitudinal scan plane illustrating measurements of the prostatic length (1) and prostatic depth (2) in centimeters in one of the study objects. The length was defined as the maximum dimension along the urethral axis and the depth was defined as the maximum dimension in the orientation perpendicular to the course of the urethra. To the left of the image, the urinary bladder is visible. Cranial is to the left of the image

Previous cadaveric studies showed a higher repeatability of prostatic measurements in the longitudinal plane, compared to the transverse plane [10]. Therefore, the measurements of the prostate gland selected for the purpose of the current study were acquired in the longitudinal plane. Effort was made to acquire optimal longitudinal orientation during the examination, in which the prostatic urethra was used as a hallmark for true sagittal orientation. The ultrasound probe was placed transversely at the level of the trigone of the bladder and moved caudally while visualizing the urethra. The urethra was followed caudally until the prostate gland was visualized, at which instance the ultrasound probe is turned 90-degree clockwise in order to obtain a longitudinal image of the prostate gland centered on the prostatic urethra. The maximal length (L) of the prostate was defined as the maximum length along the urethral axis and the maximal depth (D) was measured in the dorsoventral direction perpendicular to the course of the urethra (Fig. 1).

The measurements were recorded in millimeters with one decimal point accuracy.

### Statistics

Statistical analysis was performed by author (SH) with a PhD in Health Technology Assessment and extensive experience with statistical analysis of biomedical data. All statistical tests were performed using a free software environment for statistical computing and graphics (R 3.6.1 using RStudio 1.2.1335). For a subset of the statistical analyses, the subjects were divided into three weight categories, small dogs (< 10 kg), medium-sized dogs (10–25 kg) and large dogs (> 25 kg).

A Shapiro–Wilk test was performed to assess the normality of both the length and depth measurements of the prostate. The mean age for the three different weight categories was calculated and difference of age between the groups was tested with the Kruskal–Wallis test. The median of the prostatic length and prostatic depth for the three different weight categories were determined, as well as the interquartile range.

A non-parametric test (Kruskal–Wallis) was performed to obtain weight category-based references for both prostatic length and prostatic depth. Mann–Whitney tests were performed for comparison in between the different groups (i.e. < 10 kg vs. 10–25 kg, < 10 kg vs. > 25 kg, and 10–25 kg vs. > 25 kg), followed by a Bonferroni correction.

A linear regression analysis was performed with prostatic length or prostatic depth as dependent variable and subject weight (continuous) as independent variable.

For assessment of the second hypothesis, a correlation test (Kendall’s Tau) was performed to estimate the correlations between age in months and either prostatic length or prostatic depth.

For all statistical tests, the level of significance was set at P < 0.05.

## Results

Two dogs were excluded after data collection due to suboptimal image quality of the archived images and cine loops. The number of animals excluded before data collection was not recorded. A total of 72 dogs met the final inclusion criteria and the data sets of these dogs were included for statistical analysis. Both the small dog group (< 10 kg) and medium-sized dog group (10–25 kg) consisted of 25 animals, the large dog group (> 25 kg) consisted of 22 dogs. The average age in years for the different weight categories and the age range was as follows: 9.2 (1.8–15.8), 7.4 (1.5–14.3) and 8.1 (1.9–11) years, for small, medium-sized and large dogs, respectively. There was no significant difference between the ages of the three weight categories (P = 0.263). Within the small dog group the following breeds were represented: eight mixed breed dogs, five Chihuahuas, two Pomeranians, two Boston Terriers, two Jack Russell Terriers and one of each of Border Terrier, Miniature Schnauzer, Maltese dog, Shih Tzu, Dachshund and West Highland White Terrier. In the medium-sized dog group the following breeds were represented: six mixed breed dogs, two Labradoodles, two Beagles and one of each of Stabyhoun, Border Terrier, German Shepherd, Kooikerhondje, Cavalier King Charles Spaniel, Poodle, Cocker Spaniel, English Cocker Spaniel, English Springer Spaniel, French Bulldog, Bichon frisé, Basset Hound, Västgötaspets, Whippet and Shiba Inu. Within the large breed dog group the following breeds were represented: six mixed breed dogs, four Labrador Retrievers, two Rottweilers and one of each of Bernese Mountain dog, Boerboel, Belgian Shepherd, White Shepherd, Barbet, Staffordshire Bull Terrier, American Staffordshire Terrier, Bullmastiff, Boxer and Labradoodle. Mixed breed dogs were the most prevalent in all three weight categories.

All dogs were castrated at least 6 months before the ultrasonographic examination.

The Shapiro–Wilk test revealed a non-normal distribution of both the prostatic length and prostatic depth.

**Fig. 2:**
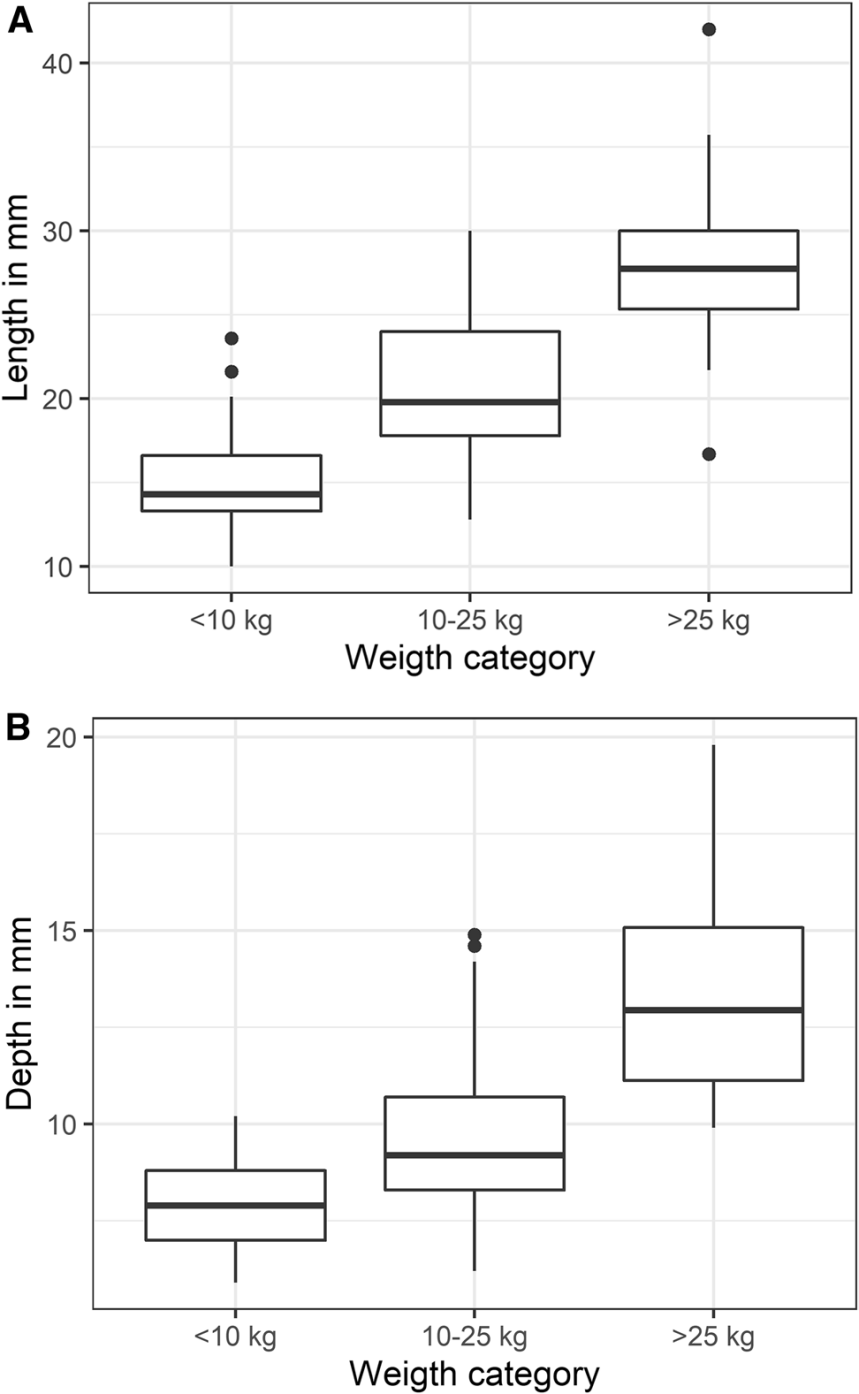
Prostatic length **A** and depth **B** measurements for 3 different weight categories. **A** Boxplot with whiskers presenting the prostatic length ( mm) assessed on ultrasonography within the three different weight categories (small dog < 10 kg (n = 25); medium-sized dog 10–25 kg (n = 25) and large dog > 25 kg (n = 22)). The prostatic length differed significantly between the three weight categories. The box represents the first and third quartile, the median is represented by the central line within the box and the whiskers represent the 1.5 interquartile range. The dots represent the outliers. **B** Boxplot with whiskers presenting the prostatic depth ( mm) assessed on ultrasonography within the three different weight categories (small dog < 10 kg (n = 25); medium-sized dog 10–25 kg (n = 25) and large dog > 25 kg (n = 22)). The prostatic depth differed significantly between the three weight categories. The box represents the first and third quartile, the median is represented by the central line within the box and the whiskers represent the 1.5 interquartile range. The dots represent the outliers

Descriptive statistics of the collected data, including median and interquartile range, are graphically illustrated in Fig. 2A, B

Both prostatic length and prostatic depth varied with weight category (P < 0.001). All weight categories differed significantly from each other (P < 0.05).

The results of prostatic length and depth measurements are depicted in a boxplot with whiskers for the three different weight categories in Fig. 2A (prostatic length) and Fig. 2B (prostatic depth). Figure 2A, B illustrate significantly differing median linear prostatic dimensions. The figures do however illustrate a significant overlap between prostatic depth measurements between the small-dog group and medium-sized dog group. For prostatic length two subjects had values above the 1.5 interquartile range in the small dog group and for the large dog group one subject had a value higher than the 1.5 interquartile range and one subject had a value lower than the 1.5 interquartile range. For prostatic depth two subjects had values above the 1.5 interquartile range, both of which were in the medium-sized dog group (Fig. 2A, B).

**Fig. 3:**
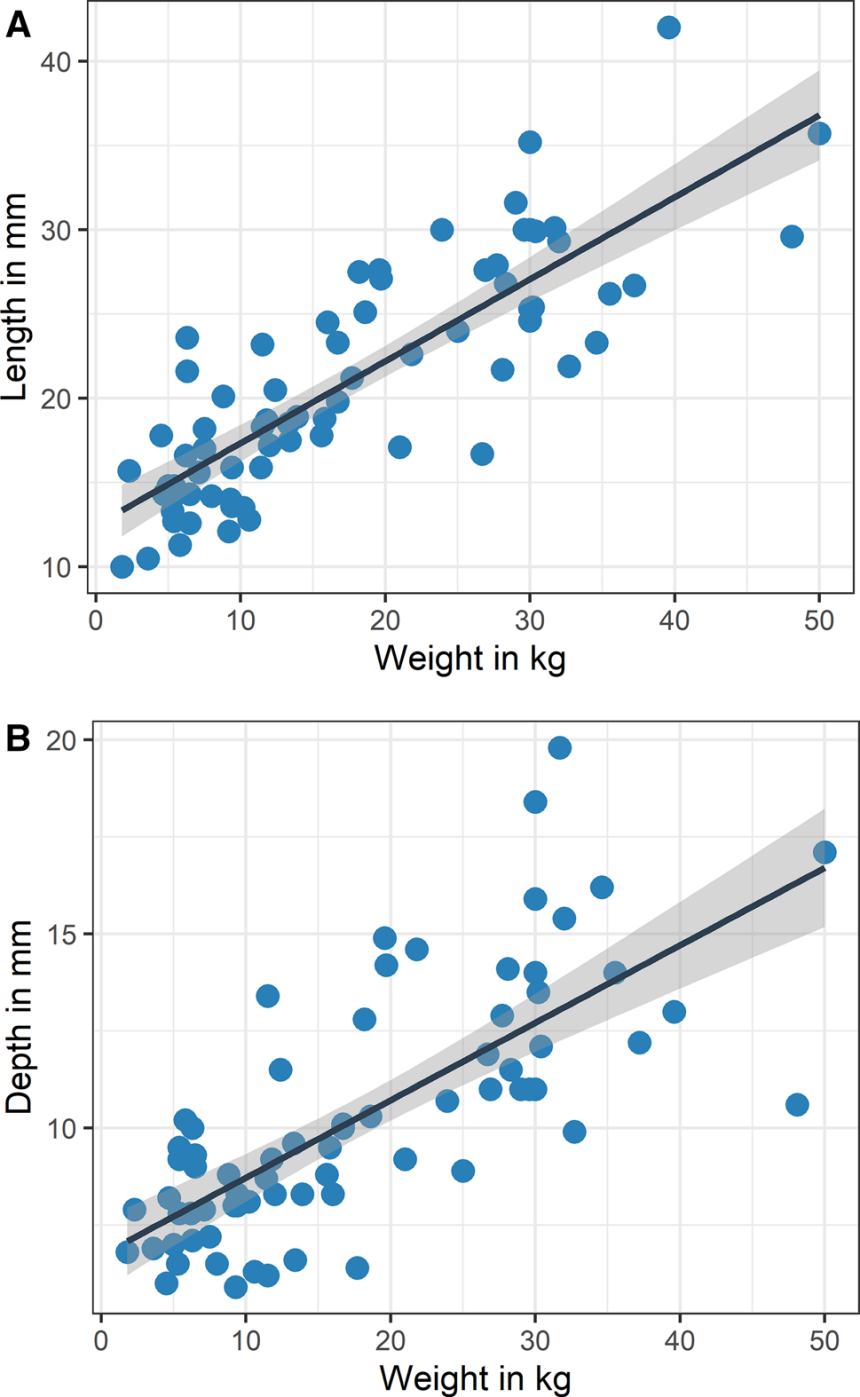
Prostatic length **A** and depth **B** measurements with weight as continuous variable. **A** Linear regression analysis of prostatic length versus body weight in 72 castrated adult canines assessed on ultrasonography. The gray shadowed region surrounding the black solid line represents the 95% confidence interval. **B** Linear regression analysis of prostatic depth versus body weight in 72 castrated adult canines assessed on ultrasonography. The gray shadowed region surrounding the black solid line represents the 95% confidence interval

With increasing body weight, linear dimensions of the prostate gland increased. Linear regression analyses of the data set showed a significant positive correlation between subject weight and prostatic length, and between subject weight and prostatic depth, with an r^2^ of 0.69 and 0.53 respectively (Fig. 3A (prostatic length) and B (prostatic depth); P-values < 0.001).

The regression formula estimated for determining prostatic length (P _L_), with weight in kg (W) as a predictor variable, was: ${\text{P}}_{{\text{L}}} \, = \,{12}.{47}\, + \,({\text{W}}\, \times \,0.{49})$.

For prostatic depth (P _D_) the following regression formula was estimated:$\left( {{\text{P}}_{{\text{D}}} } \right)\, = \,{6}.{7}\, + \,({\text{W}}\, \times \,0.{2}0)$.

Kendall’s Tau rank test showed significant correlations between weight and the two prostatic dimensions (Kendall’s rank correlation tau length 0.635 and weight 0.559, P < 0.001).

Kendall’s Tau rank test showed no correlations between age and the two prostatic dimensions (Kendall’s rank correlation tau length -0.120 and weight -0.078, P > 0.100).

## Discussion

This is the first prospective, observational study assessing prostatic dimensions in castrated adult canines, with ultrasonography. The results of the present study provide references of prostatic dimensions, which show a clear positive correlation with the dog’s body weight. Both the measurements of prostatic length and depth, in the longitudinal plane, showed a significant increase with increasing body weight. In current clinical practice, during the ultrasonographic abdominal examination, the prostate is assessed based on subjective characteristics, such as shape, size and homogeneity [15, 16]. Therefore, evaluation of the prostate is highly operator dependent and interpretation of possible abnormalities can thus vary between operators. This weight-based prostatic size reference will aid both radiology specialists and general practitioners with a comprehensive, objective parameter and can function as an add-on to the overall assessment of the prostate gland of castrated dogs. However, care must be taken to assess not only size, but also factors as homogeneity, contour, periprostatic fat and regional lymph nodes. The present study focused merely on measurements in the sagittal plane, as earlier studies showed this to be the most reproducible measurement [10], and not on volumetric measurements or measurements in additional planes, which may be more sensitive for the detection of prostatomegaly.

In the current study, a linear correlation between body weight and prostatic dimensions was illustrated. From the available data set, formulas were derived in order to calculate a prostatic length and depth reference based on the dogs’ body weight. Three weight-based categories were chosen, in order to represent the vast variety of different sizes within this species, while also keeping the reference a practical tool for daily use.

The results of the current study are in line with a recent study comparing volumetric assessment of the prostate gland in 57 intact and 37 castrated canines, as measured using the slice addition technique in CT [18]. A study regarding linear prostatic dimensions of 62 castrated dogs, as measured on computed tomographic images, revealed a positive association between body weight and prostate dimensions, also in line with our current findings [12]. The work of Atalan et al. [10] failed to illustrate a relationship between prostatic size and weight of the dog in castrated animals, which was attributed to the very small sample size of castrated dogs (17 dogs were present in the castrated dogs group) in that study.

However, another recent study conducted by Spada et al. [11] provided noteworthy, contradictory results. This study primarily addressed contrast-enhanced ultrasonographic findings in normal prostates of castrated canines, and found no correlation between prostate volume and body weight. In this study the prostate volume was calculated based on three linear dimensions of the prostate gland as a continuous variable. A possible explanation for these contradictory results could be attributed to the multiple variables (prostatic length, height and width) contributing to the prostatic volume which is then compared to body weight in the study by Spada et al. [11], whereas in the current study (as well as in the mentioned studies by Haverkamp et al. [18] and Delaude et al. [12]), the different linear dimensions are compared separately with the dogs’ body weight. Instead of a single measurement, with a single possibility for measurement error, each of the multiple measurements poses a risk for an additional measurement error. This mechanism can potentially increase the possibility to obscure a possible true relation between prostatic dimensions and body weight. Also, the effect of body condition score was not evaluated in either the current study or the referenced studies, but could be of influence on the measurements. Dogs of the same body size but with different body condition scores could be classified in different weight categories, thus influencing the calculated references. Future anatomical studies, preferably with larger sample sizes, are warranted to investigate these contradictory imaging findings. To address differences in body condition score, references based on parameters other than weight could be of value (i.e. aorta diameter, vertebral length etc.).

In our current study, significant differences in prostatic dimensions were found between weight categories. However, a few outliers were reported as well. The two most notable outliers were found in the large dog group and consisted of the prostatic length measuring 42 and 16.7 mm respectively. The first dog was one of the largest dogs in our study population, with a body weight of 39.6 kg. The dog with a prostatic length well below the large dog groups’ median prostatic length (16.7 mm) was in fact relatively small compared to the other dogs in the group and only marginally fell into the large dog group with a body weight of 26.7 kg. We would suggest to relate these abnormal prostatic lengths to the relative position of these individuals within the large dog group. As per inclusion criteria, the prostates of both individuals were interpreted to be normal, based on the assessment by the radiologist or radiology resident performing the ultrasonographic examination, in accordance with the current standard of practice. Due to the relative invasiveness and moderate technical difficulty, cytological samples of the prostates of the included individuals were not included in the study design. Thus, disease cannot be completely excluded as a cause of the relatively large prostatic length in the mentioned individual, as cytology and/or histology is considered the gold standard in diagnosing canine prostatic disease. A too small prostatic length is not expected as indicator of prostatic disease and therefore the second individual, with a relatively small prostatic length compared to the group mean value, would be highly unlikely to represent pathology. Furthermore, prostatic depth dimensions were within the reported reference for their weight category for both individuals, supporting lack of pathology as a cause of the abnormal prostatic length. In clinical practice, these outliers, and thus overlap between size ranges of normal vs. abnormal prostates, should be kept in mind, in order to prevent over-interpretation of prostatic disease. Comparison of the different prostatic dimensions with the subjects age revealed no relationship between these parameters, which was in line with what was expected based on both previous studies and clinical findings in daily practice [10].

Several limitations to the current study design exist. First of all, as mentioned, there was no cytological or histological confirmation that the prostates under investigation indeed were normal. Due to the study population and study design this was not feasible, and the level of invasiveness required for obtaining cytological or histopathological specimens would be unethical. Furthermore, even with the availability of cytological or histopathological biopsies, the possibility remains that a lesion is missed (i.e. false negative), especially when encountering focal pathologies (as apposed to generalized prostatic disease). The possibility of prostatic pathology within our study population cannot be completely excluded, however due to the study inclusion criteria, the authors deem this to be unlikely.

In the current study design, data collection and measurements were carried at a single point in time by a single observer. This eliminates possible interobserver variability as a cause for spread in measurements. Future study designs could further explore the interobserver variability by introducing multiple observers, and different levels of experience between observers, in order to better reflect the clinical setting. Intraobserver variability could also be assessed in future studies.

Suboptimal imaging of the complete prostate gland was one of the exclusion criteria in the current study, given that improper or uncertain identification of organ margins could result in improper measurements and thus negatively influence the validity of the presented measurements. However, in clinical practice we do encounter situations where organ boundary identification can be problematic (e.g. obese patients, prostates with an intrapelvic position). Distinguishing the prostate gland from periprostatic fat and the urethra can be challenging, especially for less experienced ultrasonographers [25]. Techniques have been described to better visualize the prostate gland, e.g. transrectal ultrasonographic examination or infusion of the urinary bladder with saline, which can surpass some of the described inherent patient factors [17]. In the current study these techniques were not applied, as it would increase the invasiveness of the procedure. In clinical practice however, these techniques could be used when justified by the clinical presentation of the patient at the discretion of the clinician.

When exploring possible future aims of research, thought should be given to the specific ultrasonographic findings in canine prostatic neoplasia in castrated males, compared to normal prostates. Exploring these differences can aid further into prioritizing differentials for ultrasonographic findings, and can thus support the clinician into putting resources to appropriate follow-up diagnostics. For the present study this was not feasible, as the number of neutered male patients presenting to our clinic within the study timeframe, with confirmed prostatic neoplasia, was too small to make valid comparisons.

## Conclusions

The current study provides a first comprehensive reference for the ultrasonographic dimensions of the prostate gland in adult castrated canines and demonstrates a clear positive relationship between body weight and prostate size in this group of individuals.

## Data Availability

The datasets used during the current study are available from the corresponding author on reasonable request.
